# Future Intentions of Adolescents towards Physical Activity, Sports, and Leisure Practices

**DOI:** 10.3390/healthcare12010066

**Published:** 2023-12-27

**Authors:** Mᵃ Alejandra Ávalos-Ramos, Mᵃ Teresa Pascual-Galiano, Andreea Vidaci, Lilyan Vega-Ramírez

**Affiliations:** Department of General and Specific Didactics, Faculty of Education, University of Alicante, 03690 Alicante, Spain; mtpg14@gcloud.ua.es (Mᵃ.T.P.-G.); av73@alu.ua.es (A.V.); lilyan.vega@ua.es (L.V.-R.)

**Keywords:** adherence, predisposition, difficulties, gender, health

## Abstract

The World Health Organization warns of alarmingly high levels of sedentary behavior in our current society. In terms of the adolescent population, a substantial percentage of them do not meet the global recommendations for weekly physical activity. Therefore, this qualitative study aimed to analyze the predisposition and future preferences for physical activity and sports (PAS) and leisure practices of 142 adolescents, as well as identifying the challenges and proposed improvements, while considering gender differences. The participants’ reflections were gathered through semistructured interviews and focus groups and subjected to an inductive analysis. The main findings indicate a high intention to engage in future PAS and leisure activity. Individual sports were predominantly mentioned by girls, while team sports were mainly favored by boys. Additionally, boys expressed a preference for technology-related leisure activities, whereas girls leaned towards artistic activities. The participants emphasized the necessity to renew sports and recreational facilities and to adapt to the range of activities offered. At the same time, they acknowledged that reallocating economic resources would improve the quality of sports and health-care services. Consequently, institutions should tailor their programs of PAS and leisure activities to align with the preferences, ages, and genders of the youth population.

## 1. Introduction

The World Health Organization (WHO) [1] warns of exceedingly high rates of sedentary behavior in our present-day society. Approximately 80% of adolescents between the ages of 11 and 17 fail to meet the global recommendations for weekly physical activity and sports (PAS). These statistics are alarming, given that adolescence is characterized as a critical period in the establishment of a lifestyle that may extend into adulthood [2].

The Spanish Association of Pediatrics [3] reports that 45% of young people aged 15 to 24 engage in minimal or no PAS. Consequently, a significant portion of the adolescent population would be missing out on the numerous benefits that sustained participation in PAS could offer. These benefits are acknowledged worldwide and encompass various factors, including a decreased risk of different health conditions, enhanced mental well-being, and an overall improvement in the quality of life [1,4,5]. Nevertheless, as previously mentioned, contemporary society is marked by its sedentary nature. The Spanish Foundation of Nutrition presents a set of challenges closely linked to sedentary lifestyle habits [6]. These challenges predominantly stem from excessive technology use, surpassing the recommended maximum screen time. It has been observed that 59.8% of adolescents spend a significant amount of time on sedentary screen-based activities, including television, video games, and internet use for more than 2 h per day.

In Spain, these habits indicate that 60.6% of the male population and 56.8% of the female population aged 13 to 17 are exposed to more than two hours of daily electronic device use. Furthermore, there has been a noticeable decline in active commuting to educational institutions [6].

It is essential to emphasize that adolescence is a period during which individuals often exhibit indifference and slight disinterest in various aspects of life, including sports activities [7]. Most young people have engaged in some form of PAS until around the age of 12. The day-to-day practice of PAS at this stage focuses on activities conducted within the educational institution, extracurricular engagements, sporting contexts such as clubs, and certain leisure moments [8].

Indeed, adolescents encounter certain difficulties when engaging in PAS. Some of the barriers include issues related to the incompatibility of activities, the lack of time, or demotivation due to the routine and imposed nature of the practice [9,10]. Thus, boredom during physical education (PE) classes could serve as a strong predictor of adolescent sports abandonment [2]. In this regard, personal reasons have been identified as contributing to adolescents disengaging from sports activities. Among the primary factors, laziness, the desire for free time for social interaction, and an inclination towards the use of technology stand out prominently [11].

On the other hand, there are additional factors associated with the environment of young individuals that could influence their engagement with PAS. Family sporting habits or having a group of friends involved in PAS can serve as motivating factors for participation [12,13]. Additionally, barriers linked to family economic status may limit opportunities for PAS, especially in institutionalized settings [14]. There appears to be a growing trend in income-related inequalities [15]. Furthermore, factors such as environment of residence, public sports offerings, and availability of facilities or resources could contribute to the continuity and satisfaction of young individuals regarding PAS [7].

It appears that the leisure preferences of contemporary society have evolved, necessitating an action plan centered on the basic needs of the population. This approach directs efforts toward the provision and practice of PAS to counteract sedentary behaviors [16]. Apart from these considerations, there are additional factors linked to psychological preparedness. Notably, gender differences in the practice of PAS have been observed, alongside the examination of variables such as personality, motivation, and beliefs related to the sports activity domain [17].

Girls and female adolescents tend to exhibit lower levels of PAS, both in terms of frequency and intensity [18]. These observations are supported by data from Spanish sports federations, where female licenses represent approximately 25% in contrast to the 75% held by males [19]. A similar trend is evident in the European context, with boys devoting more time to PAS while girls allocate their time to sedentary, socializing activities [15,18].

Gender stereotypes associated with sports practices, often based on a masculinized image, may contribute to potential differences between males and females [18]. However, there are also gender stereotypes associated with sports typically considered “feminine” when practiced by males. Nevertheless, these lines are becoming increasingly blurred and less noticeable considering the inclusive values embraced by contemporary society [20]. In this regard, a gendered model of sports in which the offerings do not align with individual needs could lead to disinterest in sports during leisure time. A systematic review carried out by Boraita et al. [21] emphasized the importance of the educational setting, specifically PE sessions, in interventions designed to promote PAS among adolescent girls. Establishing an enjoyable environment and incorporating diverse, noncompetitive activities could increase participation and address the requirements associated with physical activity engagement.

Thus, there is a need to promote PAS and leisure activities that resonate with participants, fostering a disposition toward leisure time engagement and ultimately achieving a healthy lifestyle from a young age.

Therefore, the objectives set forth in this research were twofold. The first was to analyze future intentions and preferences regarding engagement in PAS and leisure activities among a group of adolescents, while identifying the challenges they encounter in this domain. The second was to explore the improvement suggestions put forth by adolescents in response to the identified issues, all within the framework of a gender perspective.

## 2. Methods

The qualitative approach in this case study is ideal for delving deeply into the experiences and perspectives of adolescents regarding the stated objectives. The narrative reflection on the present and future of PAS and leisure activities by the young participants will enable the analysis of their lived experiences, and based on these experiences, the identification of new insights that may alter previous or current practices and guide future actions [22]; in this instance, within the municipality in which they reside.

Following Clandinin et al. [23], we conceptualize this research as a narrative thinking process, particularly based on the interaction between adolescents and the specific situations where experiences related to the practice of PAS and leisure unfold. Consequently, this study represents a form of reflective practice that enables the analysis of participants’ life experiences within their own contexts and specific situations, thereby generating novel insights.

### 2.1. Sample

A deliberate choice was made to include adolescents from a high school within a municipality in the Valencian community, primarily due to the low involvement of this demographic in PAS and leisure activities. Consequently, this preliminary investigation encompassed a specifically selected group comprising 142 adolescents (72 females and 70 males) in their first and second years of compulsory secondary education whose ages ranged from 11 to 13 years (with an average age of 12.49). The students voluntarily took part in the study, having obtained prior consent from their legal guardians. This study has obtained the approval of the Ethics Committee of the University of Alicante (UA-2020-09-01).

The coastal municipality in the Valencian community (Spain) where the analysis was conducted has nearly 30,000 inhabitants [24], with tourism and construction being key pillars of its economic activity. Education is provided through public primary and infant education schools, as well as secondary education institutes. In terms of cultural and social facilities, the municipality possesses an auditorium, an open-air theater, and exhibition halls. Additionally, it has a municipal library and a social center that includes the Youth Information Service. Moreover, there are municipal sports facilities, two covered sports pavilions, a court for the practice of local sports, two artificial football fields, tennis courts, and a municipal swimming pool under construction. This municipality offers a diverse program of PAS and leisure and provides subsidies for economically disadvantaged families and other family discounts.

### 2.2. Instrument

For data collection, two main tools were used: semistructured interviews in written format and focus groups. Writing and storytelling are a form of research, especially about oneself and one’s situation. Through narration, whether oral or written, we can understand how we construct the world [22].

In this study, the semistructured interview consisted of two open-ended questions in which each participant could express their current thoughts and reflect on the future regarding their interest and engagement in PAS and leisure activities in their community. At the beginning of the interview, demographic questions were presented in three dimensions (academic year, gender, and age), and then the two questions central to this research were explored:

1. What PAS and leisure activities would you like to engage in within your municipality in the immediate future?
2. What challenges do you encounter in your municipality when it comes to participating in PAS and leisure activities?

On one hand, written interviews were conducted with the support of two expert researchers in qualitative research. The interviewers accommodated the available schedule of the school staff, with the interviews taking place in classrooms. The average duration per grade and participant was 40 min. Anonymity of the participants was always ensured. The purpose of the interview was explained, and students were encouraged to express their perspectives, experiences, and needs related to PAS and leisure activities, emphasizing the importance of their contributions.

On the other hand, to address the research objective of understanding the improvement proposals that adolescents consider necessary for their municipality in terms of PAS and leisure activities, in their free time, the qualitative technique of focus groups was employed. In a focus group, opinions can be expressed and exchanged in an everyday context, with the sample not being isolated from the context. Additionally, it allows for corrections of opinions by the group that are “inadequate, not socially shared, or extreme, as a means of validating statements and perspectives” [25]. The focus group was employed for its flexibility, adaptability, and its ability to gather multiple perspectives, providing a deep understanding of a theme or situation [26]. The instrument consisted of two topics:

1. The engagement in PAS and leisure activities in their municipality: weaknesses.
2. Improvement actions to contribute to your city council regarding the practice of PAS and leisure activities.

For this research, two focus groups were formed, each consisting of 10 participants, with a moderator for each group. The moderator was responsible for posing the questions and restructuring the inquiries as the conversation progressed. Participant distribution was heterogeneous in terms of gender and academic year, allowing for a diversity of perspectives. The focus groups took place during the school day, with two rooms in the educational institution arranged to make the students feel comfortable and relaxed in their participation. The duration of the focus group sessions was 45 min each, and they were structured as follows:

1. Introduction.
2. Brief overview of the topic for discussion.
3. Guidelines for directing the youth, emphasizing the importance of participation, and listening to others.
4. Focus group questions.

### 2.3. Procedure and Data Analysis

To arrange the conducting of written interviews and focus groups, initial contact was made via email with the sports department of the town hall of the municipality under study. Subsequently, phone contact was made with the technician responsible. During the initial conversations, the research objective and its justification were presented. After obtaining their consent to participate, the strategy, date, location, and time for data collection were specified. Following the acquisition of the necessary permissions from the educational institutions involved and subsequently authorization from the legal guardians of the adolescents, the data collection phase commenced. This phase took place over a two-week period between November and December of the 2022–2023 academic year.

The information from the semistructured interviews was collected in written format, while the information from the focus groups was recorded in audio with the attendees’ consent. At the end of this process, the data from the focus groups were transcribed manually into Word format for the corresponding analysis. Each researcher conducted several individual readings of the obtained information. Once this phase was completed, the researchers convened to discuss the collected information and draft the coding map with the agreed-upon classification of the information. All of this was undertaken following the inductive content analysis of both the interviews and the focus groups. Subsequently, the initial connections, categories, and codes were established [27].

**Figure 1:**
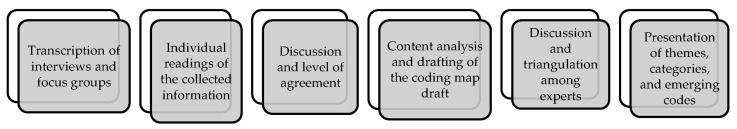
Phases of the research content analysis process: own development.

**Figure 2:**
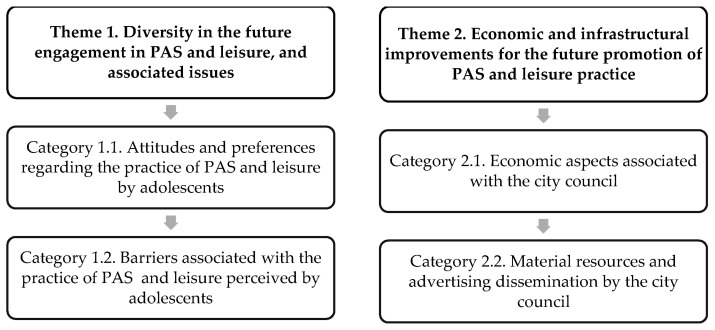
Themes and emerging categories of the research: own development.

The final descriptive and inferential codes were created and validated through a triangulation and discussion process involving two university professors specializing in PE and a high school PE teacher. After this process (Figure 1) emerged the two main themes and the categories (Figure 2). The first theme, based on the adolescents’ experiences and perceptions, determines their predisposition and future preferences for engaging in PAS and leisure activities, as well as the difficulties they currently encounter in these activities. On the other hand, the second theme presents the potential improvements proposed by young people for addressing the practice of PAS and leisure activities in their municipality from their perspectives.

Subsequently, the data were processed using the qualitative analysis program Analysis of Qualitative Data 7 [28], through the content analysis technique [27]. The software also allows for the integration of quantitative analysis with qualitative data. Therefore, in the results, we will present the percentage of the absolute frequency (%AF) to represent qualitative content. Additionally, we will include the most significant excerpts from the narrative text. The AF refers to the number of occurrences that appear concerning a specific concept.

## 3. Results

The results are presented based on the emerging themes of the research.

### 3.1. Theme 1: Diversity in the Future Engagement in PAS and Leisure, and Associated Issues

The findings in this section relate to the attitudes and future preferences of adolescents regarding engagement in PAS and leisure activities, as well as the current difficulties they encounter in the activities offered by their town hall, differentiated by gender.

#### 3.1.1. Attitudes and Preferences Regarding the Practice of PAS and Leisure by Adolescents

Our results show that the predisposition of adolescents towards future engagement in PAS is high (86.03%), with a slightly higher inclination among boys (87.28%) compared to girls (84.61%). Additionally, albeit to a minimal extent, some expressions of indecision regarding future participation in physical sports (9%) are evident, with girls being slightly more undecided compared to boys (10.57% and 7.62%, respectively). Furthermore, a minority of young people indicated indifference or no predisposition towards future engagement in physical sports (4.97%), with no notable gender differences.

Regarding the type of PAS that young people would be interested in engaging in, there is a greater predisposition towards individual sports (49.73%), with girls showing a stronger preference for them (65.90%) compared to boys (35.92%). In this regard, individual sports that were mentioned in the narratives with higher frequency were cycling, skating, and swimming. In the case of boys, cycling was predominant, while girls leaned predominantly towards skating. Despite the positive implications of peer socialization in adolescence, our participants did not consider the social factor significantly when expressing their future intention to engage in PAS. Instead, they leaned towards more individualized practices. It appears that young people prefer to engage in PAS individually, without being influenced by factors beyond their control, such as coinciding with peers in practice. On the contrary, they reflect attitudes such as discipline and self-demand as motivations for engaging in physical activity: “I like individual sports because everything depends on yourself; you can practice whenever it suits you best, according to your other activities” (Student_4); “As I grow older, I would prefer more individual physical activity because it is more demanding and involves a lot of discipline” (Student_12).

In second place, there is an interest in team sports (23.56%), with boys showing a higher preference for this option (31.06%) compared to girls (14.77%). In this context, team sports such as soccer, volleyball, and basketball are the most frequently mentioned by adolescents. Regarding gender preferences, boys would lean towards playing football in the future, while girls would incline towards volleyball. As observed in the arguments of the young individuals, the future choice of PAS follows a certain stereotyped pattern concerning sports selected based on gender. This group of adolescents seems to be aware of the socialization opportunities that sports, and physical activity provide. Moreover, they appear to be sensitive to the idea that a student is less likely to abandon regular physical activity if it promotes camaraderie among peers and a sense of group identity: “The best part is playing team sports because of the camaraderie with your friends, so it’s the best way to keep my friends in the future” (Student_14); “Team sports are more fun because you compete with the rest and you also socialize, so that’s what I will try to do when I’m older (Student_11).”

Also, future physical activities related to racket sports are mentioned (12.06%), with boys expressing more interest (14.56%) than girls (9.09%). Racket sports such as tennis, badminton, and paddle tennis were mentioned. For boys, the preference for future participation in these sports is tennis and paddle tennis, while girls lean towards badminton and tennis. At the same time, they mention fitness group activities (8.37%), with boys showing greater interest compared to adolescent girls (10.67% and 5.68%, respectively). These activities are associated with the practices of weightlifting, boxing, and kickboxing. In this case, boys prefer boxing and kickboxing activities, while girls prefer weightlifting activities. These preferences for future practices reflect the presence and rise in the fitness movement associated with promoting physical activities, health, and leisure. This trend is characterized by organizational flexibility and offers multiple diverse and dynamic modalities to future users. Group activities in fitness studios can provide customization of consumption and scheduling flexibility, guiding, and meeting users’ needs based on their preferences. This can ensure adherence to physical activity practices. The adolescents in this research seem to be aware of these advantages: “I always do group activities because I never know what to do on my own. They guide me, and it’s easier and more fun. When I’m older, I’ll join a gym to continue with these activities” (Student_9); “I’ve recently started kickboxing. It’s an activity that has just been introduced in the gym and the truth is that the timetable suits me very well and I’m in the same place as my friends” (Student_107).

To a lesser extent, a group of adolescents express an interest in such outdoor activities as hiking (3.66%), and specifically, in water sports and activities (2.61%), with both being more commonly mentioned by boys (3.88%). The mentioned water activities refer to rowing, sailing, or surfing. Despite the coastal context in which the adolescents in this study live, it seems that activities in the natural environment, including those in the maritime setting, are not considered by young people for future engagement in PAS. In this regard, it is considered that the strength that the natural environment offers for PAS is not being sufficiently exploited by institutions, as adolescents barely mention these options. Additionally, this situation could be attributed to the specificity of these activities, involving the use of specific and expensive materials, and economic factors may influence these preferences for practice: “I loved the rowing activity I did with the school. When I wanted to register, my parents did not allow it because it was too expensive” (Student_55); “The nautical activities are my favorite. I really like surfing and the best thing is the social climate. I got informed that I needed my own material, but I can’t buy it” (Student_80).

To conclude this category, it should be mentioned that—albeit to a minimal extent—young people express some indecision about whether to engage in PAS in the future (9%), with adolescent girls being slightly more indecisive than their male counterparts (10.57% compared to 7.62%). On the other hand, a minority group of young people indicates indifference or no inclination towards future PAS (4.97%), with no notable differences between genders.

Regarding the predisposition towards engaging in other leisure activities (LAs) offered in the analyzed municipality, there is also a favorable trend towards them (81.29%). Girls have a higher intention to participate (83.07%) compared to boys (79.72%). Additionally, a group of adolescents expresses indecision about how to spend their free time with LAs (13.66%), and the intentions of both genders are very similar. A minority of participants (5.03%) specifically mention that they have no interest in engaging in any LAs in the future.

The LAs mentioned by the adolescents focus on technological activities (30.43%) such as video games, virtual reality activities, robotics, activities related to content creation on TikTok, or computer activities. In this type of technological activity, boys show a clear preference for video games or robotics (50%) compared to girls (9.09%), who prefer content creation on TikTok. The influence of technology on the entertainment choices of young people is undeniable. In fact, technologies themselves constitute a fundamental part of the leisure time of adolescents. In the analysis conducted in our study, we observe that females lean towards social media, which allows them to create content and share their experiences with other users. Additionally, it enables them to build their own communities with users from different geographical locations: “I use TikTok because I can show others what I like to do and how I entertain myself. Thanks to TikTok, I meet other girls who like the same things as me” (Student_70).

The analysis also reflects that males prefer technological activities more associated with gaming, character representation, and themes. Video games have historically been more oriented towards males, with a competitive nature, contributing to the perception that video games may be primarily for boys: “I like playing video games with my friends; we have competitions many weekends. Also, we always look for the latest versions” (Student_11).

On the other hand, artistic activities such as cinema, theater, or drawing (24.34%) are mentioned, with girls (36.36%) showing more interest in this type of leisure compared to boys (13.33%), who prefer writing and reading activities. Over time, certain gender stereotypes have suggested that women might be more inclined towards visual and performing arts, such as theater and drawing. However, it is crucial to understand that individual preferences, culture, education, and exposure to different experiences also play a significant role, as reflected in the arguments of young people: “Usually, those of us who don’t do sports as extracurricular activities go to music—in my case, piano. I would love to continue practicing it” (Student_37).

There are also expressions of preference for leisure activities with family and friends, such as attending concerts (21.73%), with boys (25%) being more inclined toward this option than girls (18.18%), who prefer activities such as talks or debates. From our results, it is evident that both family and friends provide emotional and affective support to young people, creating a comfortable space for sharing experiences and valuing a sense of belonging: “I enjoy it when we go on outings with the whole family, including my uncles and grandparents. We laugh a lot and have a good time” (Student_20); “I wish there were more activities that we could do across generations” (Student_43).

Creative and design-related activities such as makeup, hairstyling, or culinary activities are exclusively mentioned by girls (27.27%). Finally, activities like board games or escape rooms (10.43%) are more emphasized by boys (11.66%) “It would be cool if there were escape rooms or places like the Ateneo where people gather to play role-playing games” (Student_42). In a minority, we find some leisure preferences with a marked gender stereotype, although today the beauty industry has evolved to include products and specific lines for all genders: “We like to do makeup in our free time; if there were any activities of this kind, I would join” (Student_51).

Finally, a group of adolescents expresses indecision regarding the use of leisure time with organized activities (13.66%), with intentions being very similar between both genders. A minority of participants (5.03%) specifically indicate that they are not interested in engaging in any organized activities in the future.

#### 3.1.2. Barriers Associated with the Practice of PAS and Leisure Perceived by Adolescents

The findings associated with the difficulties perceived by adolescents in their municipality that hinder or impede the practice of PSA and leisure in their free time are presented below.

It was found that a high number of adolescents expressed some difficulty with the practice of PSA and leisure in their free time (65.43%), with boys being the ones who most frequently report this perception. Furthermore, a smaller proportion of findings indicate that some adolescents do not encounter any difficulties in this regard (24.69%), with girls being less critical in this regard. A minority of findings reflects that some adolescents do not know or do not respond to this question (9.87%).

The interviews revealed that the difficulties encountered by adolescents in practicing PAS and leisure (Table 1) are related to the municipality’s inadequate or deteriorating facilities, as well as the lack of material and human resources (43.39%). Boys are more critical in this regard (46.80%) compared to girls (40.67%). The negative perception of young people regarding the inadequate maintenance of sports facilities is often mentioned, the maintenance of which being the responsibility of the public sector or the facility owner. The association between engaging in PAS and having sufficient and well-maintained infrastructure and resources seems to be a positive predictor for promoting sports practices of the interviewed adolescents: “There is a limited number of sports courts, so there are availability and space problems” (Student_22); “There are sports fields very far from the town, and some facilities are in poor condition, so I don’t feel like practicing in those conditions” (Student_17).

Another issue identified is linked to expensive, limited, and non-age-appropriate activity offerings (27.35%). Arguments reflecting this reality are more prominent among girls (28.81%) than boys (25.53%). The adolescents indicate that the range of activities in their municipality does not align with their interests. According to their statements, PAS proposals that align more with the interests of young people could encourage greater engagement: “There is a limited offering for teenagers; they focus more on leisure sports for children, so it is not appealing to us” (Student_30). They also express that they must pay fees, which poses a financial burden on families, especially when there are several siblings in the family. The socioeconomic level of families is a determining factor in engaging in physical activity. The adolescents in this study represent family economic inequalities, with those from lower socioeconomic backgrounds potentially missing out on opportunities for physical activity. These inequalities are reflected in their arguments.

To a lesser extent, young people mention a lack of time due to academic tasks (15.09%). Girls articulate this reason more often (18.64%) compared to their male counterparts (10.63%). For adolescents, especially for girls, tasks such as homework, attending academies for reinforcement of learning, or taking classes with private tutors are barriers that limit the practice of PAS and leisure. However, almost no adolescent acknowledges that one of the problems in this regard could stem from a potential lack of personal organization and planning when juggling their various tasks: “I have a lot of homework and exams that take up a lot of my time to do anything else” (Student_46).

Finally, some personal issues such as insecurities, lack of confidence, laziness, or family-related issues (14.15%) also act as difficulties impeding engaging in PAS and leisure in their free time: “My parents believe in myths, like if you do sports like lifting weights, you will stop growing“(Student_6); “My father cannot take me to basketball. He doesn’t have time because he is working” (Student_50).

It seems that the interviewed boys (17.02%) are the ones who perceive insecurities, a lack of interest in PAS, or attitudes like laziness towards leisure time more than girls (11.86%). All these issues perceived by young people could be associated with the abandonment of engaging in physical activity and sports. The narratives of youth reflect a negative self-perception, insecurities, and a lack of tenacity and effort towards physical activity “I find it hard to exercise because I am very lazy” (Student_19); “I am not interested in sports, and it doesn’t appeal to me. Besides, I am very bad at it. My endurance level is very low. I am not athletic at all” (Student_17).

Additionally, in the interviews, a smaller group of young people emerges who state that they do not find any difficulty in this regard (24.69%), with girls being the least critical. A minority of findings reflect that adolescents do not know or do not answer these questions (9.87%).

### 3.2. Theme 2: Economic and Infrastructural Improvements for the Future Promotion of PAS and Leisure Practice

In this section, we present the findings from the focus groups where young people addressed the issue of proposing improvements related to the provision of PAS and leisure activities in their free time, considering the challenges they identified. Notably, there were no gender differences in the contributions—the proposals were consistent across all participants.

#### 3.2.1. Economic Aspects Associated with the City Council

**Figure 3:**
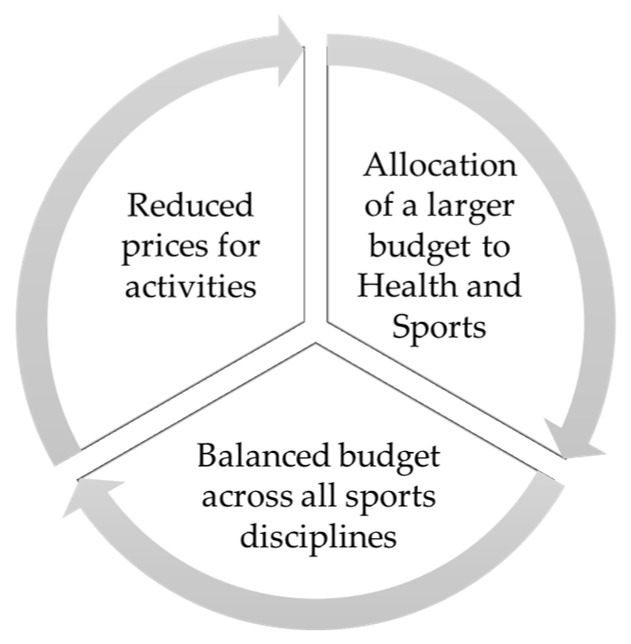
Economic improvements associated with the city council from the perspective of young people.

Primarily, as mentioned in Figure 3, the adolescents suggested that the municipality should allocate a larger budget to health and sports: “What we see is that a lot of money is allocated to unnecessary things, and it should be directed more towards health and sports” (Student_20). Concerning sports, they emphasized the need for a more balanced distribution of funds across all sports disciplines, not just favoring the more popular ones, such as football. Adolescents perceive inequalities associated with the money allocated to certain types of sports activities: “We think that some sports activities should be free or more affordable” (Student_12).

From the arguments of the young people, it is clear that efforts to promote PAS in the municipality should be directed, as much as possible, to the local community, which should provide the infrastructure and means for engagement. Therefore, adolescents can perceive how public financial support influences the practice of PAS beyond other influential factors such as family or friends: “I think that some proposals could be promoted by public institutions, for example, the Sala Jove among other spaces, in addition to offering PAS for leisure and recreation at zero cost” (Student_9).

At the same time, regarding the financial aspect, the adolescents proposed evaluating the prices of municipal activities, making them more affordable, and even considering cost-free activities or reductions for families in vulnerable situations within the municipality: “For example, I would suggest promoting the creation of public sports facilities with affordable costs for everyone or, at the very least, for those with lower incomes” (Student_10). Large families might be affected when it comes to enrolling their children in extracurricular activities. According to the perception of the young people, there is a concern about the high costs of these activities, especially when more than one family member wants to participate in PAS and LAs that involve a monthly fee. The youth suggest the possibility of accessing various scholarships or resources that ease the family’s financial burden. Examples they point out include the municipality providing assistance with registrations or for sports equipment and materials: “As a solution to the money issue, scholarships for equipment and free sports registration could be provided for the most disadvantaged individuals” (Student_8).

#### 3.2.2. Material Resources and Advertising Dissemination by the City Council

**Figure 4:**
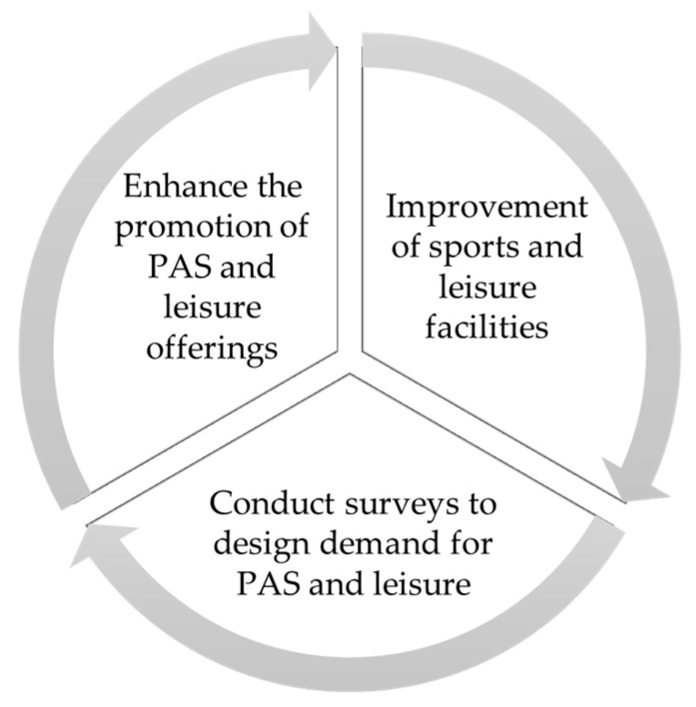
Action proposals associated with improvements in infrastructure and dissemination.

On the other hand, as presented in Figure 4, the focus groups also mentioned, as an action plan, the improvement of sports and leisure facilities by investing in their maintenance and renovating existing sports facilities. This would ensure good conditions and safety for their use. The young people even suggested the possibility of creating new infrastructures such as a public gym, the opening of a public swimming pool, or the upgrading of equipment and technology to align with current trends in fitness and sports: “Another proposal that comes to mind, and I think it’s easy to solve, would be to improve the cleanliness and access to existing facilities and explore the possibility of creating new ones” (Student_2); “As a proposal, I would consider creating or installing facilities for other sports that we quite like, such as skateboarding” (Student_17).

Additionally, our analysis suggests that the municipality should offer a more flexible schedule that adapts to the different routines and lifestyles of the community, covering various time slots (morning, afternoon, evening): “Many times, we find that the facilities are closed when we can go” (Student_9).

At the same time, the young people mentioned the possibility of implementing effective strategies to increase participation in PAS and LAs. Some of their ideas include conducting annual surveys and offering activities based on the youth demand. Adolescents suggest collecting their opinions to understand their preferences and changing needs, thus adjusting the offerings to the demand. This ensures that the activities are attractive and in line with their interests: “I would suggest improving the offerings tailored to our age and interests, aligning them with our preferences” (Student_5): “There are only activities for small children and of a low level” (Student_18): “In some activities, like the gym, they don’t let us in because we are under 14, so they could take this into account” (Student_16).

The adolescents in this study also suggested promoting, for example, through educational talks, the physical, mental, and social benefits that engaging in physical activity and sports can bring to the young and adult population. They proposed offering open days to allow the community to experience the available activities free. Similarly, this would enable trying out a new activity before committing to it: “I think they can enhance the promotion of activities through open days and information through educational institutions” (Student_16).

Other mentioned proposals refer to improving the dissemination of information about physical activity and leisure offerings and associated news, for example, through social media platforms such as TikTok or Instagram. Essentially, it should leverage platforms popular among young people to promote events, share success stories, and keep the community informed, and similarly, regularly publishing news, offers, and participation opportunities in other media outlets. The young people in this research are aware of the potential of these tools for improving information dissemination: “The information is not very clear or accessible. We have to search for information to know where to do it” (Student_9); “We would like to find information through social media, like Instagram” (Student_15); “They should be promoted with posters in central locations like schools or the TRAM” (Student_4).

## 4. Discussion

The research aimed to analyze the predisposition and future preferences for the practice of PSA and leisure activities in a group of adolescents in a Spanish municipality, as well as to identify their practice difficulties and proposals, all from a gender perspective.

The most prominent findings of this case study reveal that a high percentage of adolescents wish to engage in PAS and leisure activities in their free time in the immediate future. In the case of boys, the intention for future PAS and leisure activities is greater compared to their female counterparts, as reflected in other studies conducted in Spain [21,29,30], as well as in other countries, such as a recent study conducted in Argentina [31]. Conversely, girls express a higher preference for participating in leisure activities like movies, theatre, or drawing, unlike their male peers who prefer sports activities. Despite recent data from the WHO [1] showing a sustained downward trend in PAS and leisure activities among young people, notably during adolescence, there are several predictors that can ensure future PAS and leisure activity participation in young people during leisure time. In this regard, various studies have examined the importance of the intention to be physically active, the significance of motivation when initiating a behavior, and prior attitudes when it comes to maintaining PAS and leisure activities over time [30,32]. In our study, the predisposition to be active is highly relevant overall, as the strongly positive intention expressed by adolescents to engage in PAS and leisure activities can be a good predictor of their motivation for this activity, as demonstrated over the years [30,33,34,35,36]. However, this favorable predisposition does not seem to be a sufficient guarantee for maintaining the intention to engage in such practices over time and developing this, as evidenced by the published data on sedentary behavior in the young and adult population [1].

Regarding the type of PAS and leisure activities that aligns with their preferences, adolescents show a greater preference for individual sports in general, with girls being the primary proponents. Adolescent girls prefer activities such as dancing, rhythmic gymnastics, or skating, while boys tend to choose team sports like football, handball, and racquet sports. The results of our study are consistent with those of Peral-Suárez et al. [18] in a group of Spanish adolescents and with findings from other research carried out in Canada involving child populations [37]. Individual sports continue to be the preference for females from a young age, an aspect that should be considered in the design of sports activities, both in formal education and in extracurricular PAS and leisure activities offerings.

Even though this research was not addressed directly through PE, this subject and its teachers are crucial factors and potential predictors of extracurricular PAS practice and the development of lifelong physical activity habits. Numerous studies support how students’ perception and participation in PE classes can affect their motivation and interest in PAS practice based on the satisfaction of their experiences or the focus on fun and cooperation in these classes [38,39,40]. However, it is essential to consider gender in this context because girls often exhibit more resistance to the PE subject due to feelings of incompetence and a low self-concept, among other reasons [41]. Therefore, PE teachers and their subject have the opportunity to positively influence adolescent populations [42], particularly in countries like Spain or the United Kingdom, where PE is compulsory until the age of 16. In this context, the voices of younger students are crucial in shaping both the PE curriculum and extracurricular activity offerings for leisure time. PE teachers can serve as role models for physically active and healthy lifestyles among adolescents. The approach adopted in most European countries, Australia, and New Zealand, which links extracurricular practice with the PE subject, can contribute to fostering sustained PAS engagement throughout adulthood [43].

When we examine the results regarding preferences for other leisure activities, males tend to prefer technological activities, especially video games, while females lean towards artistic activities related to creativity and design. This research highlights the persistence of stereotypical beliefs and gender role expectations when it comes to preferences for leisure activities, professional pursuits, and even household responsibilities. Despite advances in gender equality, stereotypes regarding preferences for leisure activities, occupational choices, and domestic roles persist, with women and men still tending to align themselves with gender-based stereotypes [44,45].

### Challenges and Improvement Proposals for Physical Activity and Leisure Practice According to Adolescents

While the adolescents in our study acknowledge having future intentions to participate in the sports and leisure offerings in their municipality for their leisure time, they also express and identify a series of obstacles that limit or generate indecision when it comes to continuing with the municipality’s proposals.

Regarding gender, boys are the most critical in these matters and encounter more difficulties. Perhaps this is because the male population tends to be more active in the practice of physical activities and sports [39].

Among the issues most highlighted by adolescents are problems associated with infrastructure and material and human resources, the high costs of activities, or the lack of leisure activities that align with their interests. These findings are consistent with studies conducted by López-Castedo et al. [46] and Peral-Suárez et al. [18]. In this regard, there have been successful outcomes in improving these shortcomings when the management of physical activity offerings is carried out collaboratively between public and private associations. The problem lies in the fact that the private sector focuses on the use and exploitation of public facilities, while the public sector primarily pays attention to the maintenance of the facilities and additional income to compensate for budget reductions. In other words, there is no real coordination or alignment in terms of goals and existing resources [47].

In terms of gender and difficulties in engaging in physical activities and leisure during their free time, girls mention more often than their male counterparts the lack of time due to academic studies and, to a lesser extent, their personal insecurities [48]. On the other hand, boys primarily highlight laziness or lack of competence in sports. These findings align with the studies by Martínez et al. [49] and Ávalos et al. [50], which conclude that a lack of time due to academic tasks and typical adolescent attitudes like laziness, lack of effort, or personal insecurities and fears are significant reasons for discontinuing physical activity.

Finally, the adolescents in this case study propose, in terms of the allocation of financial resources, the need to allocate a larger budget to health and sports in the municipality. Various studies support the importance of funding for healthy foods and beverages, health, and nutrition education, as well as support and opportunities for physical activity to foster a healthier and more active population [51,52]. The younger population in our study is aware of the importance of prioritizing these areas. For the promotion of physical activity and sports, and consequently for overall physical, mental, and social health, municipalities could consider actions such as implementing workshops targeted at adolescents on nutrition and PAS, organizing open sports events, or conducting talks aimed at young people and their families.

Financial support and investment in promoting and projecting physical activities and other leisure activities, as well as promoting physical, mental, and social health, are self-evident. However, it appears that institutions are not sufficiently addressing these issues from the perspective of adolescents. It is essential for traditional funding conditions, which often allocate greater financial support primarily to competitive sports and specific sports disciplines, to be reviewed to ensure a balance in budget distribution and to provide equal attention to health-focused physical activities [53]. In terms of the physical environment, the municipality can create or improve more space for unorganized physical activity, allowing adolescents to engage in sports with their peers. In this regard, smaller communities can offer integrated behavioral approaches that facilitate and promote the practice of PAS and LAs among parents, siblings, peers, and coaches [54].

The study has some limitations, as it did not consider perspectives on the strengths that the practice of PAS and leisure can contribute from the viewpoint of young people. Likewise, it would be interesting to explore the relationship between other genders and sporting preferences. As future lines of research, following this initial analysis, we will consider addressing a larger adolescent population from various socioeconomic levels and different age groups, as well as the analysis of other municipalities on these issues to draw conclusions that can establish action protocols and design and implement strategies that can enhance adherence to the practice of PAS and LAs from an early age.

## 5. Conclusions

Based on the results of this case study, without aiming to generalize our findings outside the analyzed context, we can conclude on the need to consider the high intention to engage in PAS and leisure activities in the future, as well as the preferences and interests of the younger population regarding leisure time. It is crucial and necessary for both public and private institutions to adapt their programs of PAS and leisure activities according to the preferences, age, and gender of the population under analysis.

Adolescents express a future intention to spend their leisure time in individual physical activities for girls and collective physical activities for boys. Additionally, in terms of other leisure activities, male adolescents show preferences for technological activities, specifically video games, and to a lesser extent, females prefer social media. It is also noteworthy that adolescent girls emphasize engaging in artistic activities.

Therefore, our study reveals the wide variety of activities that young people mention as possibilities for future practices. The challenge often lies in the lack of alignment between the offered activities and the younger population’s interests. It seems that, when designing programs, the voices of young people are not being taken into account, and the trend in launching a wide range of activities is becoming generalized without considering the age groups of potential practitioners.

The main difficulties to consider, as highlighted by adolescents, include the need to renovate and improve the quality of sports and leisure facilities, as well as the offerings of activities that they signal as expensive, reduced or not according to their needs.

As the main proposals for improvement, the young people suggest the need to renovate and enhance the quality of sports and leisure facilities and material resources in their municipality.

Additionally, the youth are aware that an improvement in the distribution of economic allocations could reduce perceived inequalities based on the type of physical activity and sports offered. This, in addition to promoting scholarships and cost-free activities for disadvantaged families and large families, could optimize the quality of sports services.

The young people in this study advocate for the use of social media to enhance the dissemination of information regarding the offerings of physical activity and leisure and the implementation of surveys to better understand their interests.

Therefore, the various stakeholders responsible for creating opportunities and situations for active and healthy leisure could use the information obtained in this research to design and offer individualized or tailored interventions or recommendations for the younger population. This could contribute to increasing participation rates of the young population in terms of engaging in physical activities and attending leisure activities organized by various institutions.

## Figures and Tables

**Table 1:** Causes of difficulties related to the practice of physical activities and leisure according to gender.

Code	Group		Girls		Boys	
	AF	%AF	AF	%AF	AF	%AF
Facilities and Resources	46	43.39	24	40.67	22	46.80
Expensive, Limited, and Incompatible Offerings	29	27.35	17	28.81	12	25.53
Lack of time due to Academic Tasks	16	15.09	11	18.64	5	10.63
Personal and Family-Related Issues	15	14.15	7	11.86	8	17.02
Total	106		59		47	

## Data Availability

Data are contained within the article.
